# Prenatal echocardiographic classification and prognostic evaluation strategy in fetal pulmonary atresia with intact ventricular septum

**DOI:** 10.1097/MD.0000000000017492

**Published:** 2019-10-18

**Authors:** Lin Liu, Hongdan Wang, Cunying Cui, Yanan Li, Yuanyuan Liu, Ying Wang, Taibing Fan, Bangtian Peng

**Affiliations:** a Department of Ultrasound; b Gene Center; c Children's Heart Center, Henan Provincial People's Hospital Cardiac Center, Fuwai Central China Cardiovascular Hospital, People's Hospital of Zhengzhou University Cardiac Center, Zhengzhou, China.

**Keywords:** congenital heart diseases, fetal echocardiography, postnatal evaluation, prenatal diagnosis, pulmonary atresia with intact ventricular septum

## Abstract

Fetal pulmonary atresia with intact ventricular septum (PA/IVS) is a rare congenital heart disease. The present study aimed to classify PA/IVS and determine the relationship between prenatal echocardiographic characteristics and postnatal biventricular or univentricular repair strategies.

A total of 51 fetuses with PA/IVS were examined from 2012 to 2019. Data on prenatal echocardiography, associated anomaly, karyotype, and outcome were collected. Two-dimensional measurements included tricuspid valve (TV) *z*-score, mitral valve (MV) *z*-score, TV/MV ratio, and ratio of right to left ventricle (RV/LV) length, whereas color Doppler measurements included degree of tricuspid regurgitation (TR), ventriculo-coronary artery communication (VCAC), tricuspid inflow duration (TID), cardiac cycle duration (CCD), middle cerebral artery pulsatility index (MCA PI), and umbilical artery pulsatility index (UA PI). Diagnostic classification was based on the development of RV and the presence or absence of VCAC. Postnatal evaluation was divided according biventricular or univentricular repair.

Of the 51 fetuses with PA/IVS, 20 were type I, 17 were type II, and 14 were type III. Only one fetus exhibited right aortic arch. The karyotype of all the fetuses was normal. Of the 28 patients who underwent postnatal surgery, 13 (46%) underwent biventricular repair and 15 (54%) underwent univentricular repair. TV *z*-score was significantly higher for the biventricular repair group compared with univentricular repair group (−1.20 ± 0.98 vs −4.33 ± 0.80, *P* = .000). TV/MV, RV/LV length, and TID/CCD were significantly higher for the biventricular repair group than the univentricular repair group (0.81 ± 0.14 vs 0.54 ± 0.09, 0.71 ± 0.11 vs 0.49 ± 0.09, 39.20 ± 3.84 vs 29.16 ± 4.58, *P* = .000). Moderate or severe TR and VCAC were significantly different between the 2 groups (*P* = .000). Gestational age, MCA PI, and UA PI did not differ between the 2 groups (*P* = .72, *P* = .36, *P* = .06). The cutoff values for the biventricular repair characteristic curves were TV *z*-score >−3.28, TV/MV ratio >0.71, RV/LV length >0.62, and TID/CCD >33.95%. The sensitivities of the TV *z*-score, TV/MV, RV/LV length, and TID/CCD were 100%, 77%, 85%, and 92%, respectively. The specificities of the TV *z*-score, TV/MV, RV/LV length, and TID/CCD were 94%, 100%, 100%, and 94%, respectively.

Fetal echocardiography was able to classify PA/IVS according to variable degree of RV and VCAC. In fetal PA/IVS, TV *z*-score >−3.28, TV/MV >0.71, RV/LV length >0.62, TID/CCD >33.95%, moderate and severe TR, and the absence of VCAC were associated with postnatal biventricular repair strategy. These findings may have implications for prenatal counseling and prediction of fetal outcome.

## 1 Introduction

Fetal pulmonary atresia with intact ventricular septum (PA/IVS) is a rare congenital heart disease. It presents as a morphologically heterogeneous lesion characterized by disconnection between the right ventricle (RV) and pulmonary artery, variable degrees of RV and tricuspid valve (TV) hypoplasia, and the absence or presence of ventriculo-coronary artery communication (VCAC).^[1,2]^ Pregnancy continuation, operation strategy, and survival rate of PA/IVS are closely related to pathological changes. A well-developed RV can sustain lung circulation and can be treated by postnatal biventricular repair. Nevertheless, an underdeveloped or inadequate size of RV requires the fetus to undergo univentricular repair postnatally. Most parents tend to choose to terminate the pregnancy if discover that their baby has univentricular circulation with a palliation strategy that ultimately requires a Fontan operation.^[3–5]^ Therefore, it is extremely important to successfully predict the outcome of fetal PA/IVS and determine which fetuses are able to undergo biventricular repair. The present study had 2 aims: the first was to identify a classification of PA/IVS and the second was to determine fetal echocardiographic predictors of appropriateness for biventricular or univentricular repair.

## 2 Methods

### 2.1 Study population

A total of 51 fetuses with PA/IVS diagnosed at the Cardiac Center of the Henan Provincial People's Hospital, China from 2012 to 2019 were studied. The maternal age was 20 to 43 (26.36 ± 5.32) years, and the gestational age was 18 to 37 (27.19 ± 5.01) weeks. The study inclusion criteria were fetuses with prenatal echocardiography and postnatal follow-up integrated data. Fetuses that did not end in a live birth and fetuses that underwent fetal intervention were used for the first aim of study and were excluded from further analyses. Fetuses included in the second part of the study were divided into 2 groups based on postnatal outcome. The biventricular repair group received a 3-ventricle repair, whereas the univentricular repair group received a single-ventricle repair. Fetuses with Ebstein's anomaly of the TV were excluded. This study was approved by the institutional research ethics board of the Henan Provincial People's Hospital.

### 2.2 Ultrasound equipment

Fetal echocardiography was measured using a Voluson E8 ultrasound system (GE Healthcare, Zipf, Austria) with a RAB4-8 probe, frequency of 4 to 8 MHz, set to ‘Fetal Cardiac’ examination mode. Pediatric echocardiography was measured using either a Philips iE33 ultrasound system (Philips Healthcare, Basso, USA) with a S5-1 probe, frequency 1 to 5 MHz, set to “Pediatric” detection mode or a GE E95 ultrasound system (GE Healthcare, Horten, Norway) with a 6S-D probe, frequency 2.4 to 8.0 MHz, set to ‘Paediatric’ detection mode.

### 2.3 Fetal heart examination guidelines

Fetal echocardiography was performed using the 2013 International Society of Ultrasound in Obstetrics and Gynecology guidelines for each study with two-dimensional, color Doppler and pulsed-wave Doppler.^[6]^ Biparietal diameter, head circumference, abdominal circumference, and femur length were measured, and gestational age was calculated for each case. Abdominal transverse plane, four-chamber view, LV outflow view, RV outflow, biventricular short axis view, 3 vessels and trachea view, aortic arch long axis view, and ductus arteriosus long axis view were obtained for each case.

### 2.4 Measurements

TV annulus: The distance between anterior leaflet and septal leaflet of TV at end diastole was measured using the four-chamber view. Mitral valve (MV) annulus: The distance between anterior leaflet and inferior leaflet of MV at end diastole was measured using the four-chamber view. TV and MV *z*-score: Measurements of the TV and MV were transformed into a *z*-score based on the derived biometry.^[7]^ RV length: The length from the root of TV to the apex of the RV cavity at end diastole was measured using the four-chamber view. LV length: The length from the root of the MV to the apex of the LV cavity was measured using the four-chamber view at end diastole. Tricuspid inflow duration (TID): Pulsed-wave Doppler displayed the time interval between the start of the E wave and the end of the A wave. Cardiac cycle duration (CCD): Pulsed-wave Doppler showed the duration of the same cardiac cycle of TID. Middle cerebral artery pulsatility index (MCA PI) and umbilical artery pulsatility index (UA PI): (peak systolic velocity − end diastolic velocity)/mean velocity. Tricuspid regurgitation (TR): ratio of TV color regurgitation area to right atrial area >30% suggested moderate or severe TR.^[8]^ VCAC: Absence or presence of communication between the RV and coronary artery could be demonstrated by adjusting to decrease the color Doppler velocity scale in order to increase the sensitivity of observing low-velocity flow. Two-dimensional and Doppler velocity was measured 3 times and the average was calculated.

### 2.5 Echocardiographic classification of fetal PA/IVS

**Figure 1:**
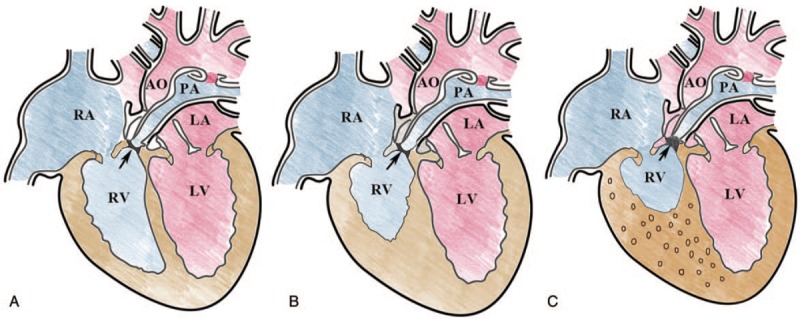
The diagnostic classification of pulmonary atresia with intact ventricular septum. (A) Type I—pulmonary atresia (*narrow*), the presence of right ventricular inlet portion, trabecular portion and infundibulum portion, the absence of venticulo-coronary artery communication; (B) Type II—pulmonary atresia (*narrow*), the absence of right ventricular trabecular portion, the presence of right ventricular inlet portion and infundibulum portion but the cavity is small, the absence of venticulo-coronary artery communication; (C) Type III—pulmonary atresia (*narrow*), the absence of right ventricular trabecular portion and infundibulum, only a small inlet portion, venticulo-coronary artery communication (*circle in the right ventricular myocardium indicates sinusoids* ). A = left atrium, AO = aorta, LV = left ventricle, PA = pulmonary atresia, RA = right atrium, RV = right ventricle.

There are 3 types of fetal PA/IVS according to the development of RV and presence or absence of VCAC (Fig. 1). Type I: presence of RV inlet portion, trabecular portion and infundibulum portion, absence of VCAC. Type II: absence of RV trabecular portion, presence of RV inlet portion and infundibulum portion but small cavity, absence of VCAC. Type III: absence of RV trabecular portion and infundibulum, small inlet portion, and VCAC. RV inlet portion can be observed in the four-chamber view from TV annulus to the cusp at end diastole. RV trabecular portion can be observed in the four-chamber view from the TV cusp to the apex of the RV. RV infundibulum portion can be observed in the RV outflow view from supraventricular crest to the subpulmonary artery.

### 2.6 Follow-up

Prenatal echocardiography, postnatal echocardiography, surgical operation, and karyotype data of the fetuses delivered after being diagnosed with PA/IVS were collected. Prenatal echocardiography, autopsy, and karyotype data of the fetuses terminated after being diagnosed with PA/IVS were collected.

### 2.7 Statistical analysis

Data are expressed as mean ± SD. Nonparametric *t* tests were performed for analyses between the 2 groups and paired *t* tests were performed for points between the 2 groups. Categorical data were analyzed using Fisher exact test. Receiver operating curves (ROCs) were used to evaluate sensitivity and specificity of the factors and areas under the curve (AUCs) for the cut-off values.

## 3 Results

### 3.1 Follow-up for all PA/IVS fetuses

**Figure 2:**
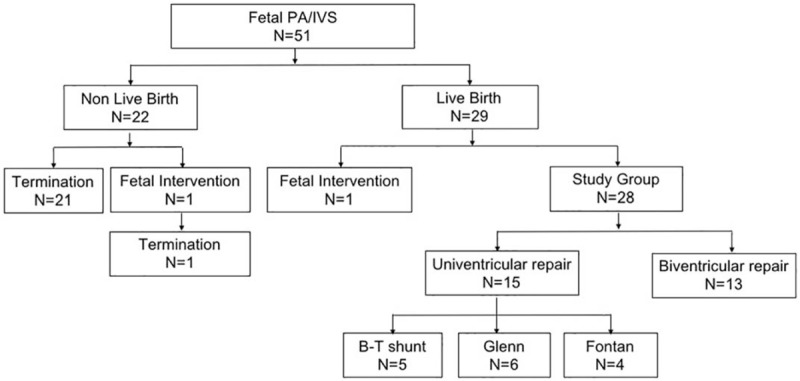
Follow-up of fetal PA/IVS in 2012 to 2019. Twenty-nine of the 51 fetuses resulted in live birth and 22 were terminated. Of the live birth fetuses, 13 out of 29 had biventricular repairs, 15 out of 29 had univentricular repairs, including 5 Blalock–Taussig (B–T) shunt operations, 6 Glenn operations, 4 Fontan operations, 1 of the 29 fetuses had a fetal intervention operation. Of the 22 terminated fetuses, one was terminated 3 weeks after fetal intervention. B-T = Blalock–Taussig.

**Figure 3:**
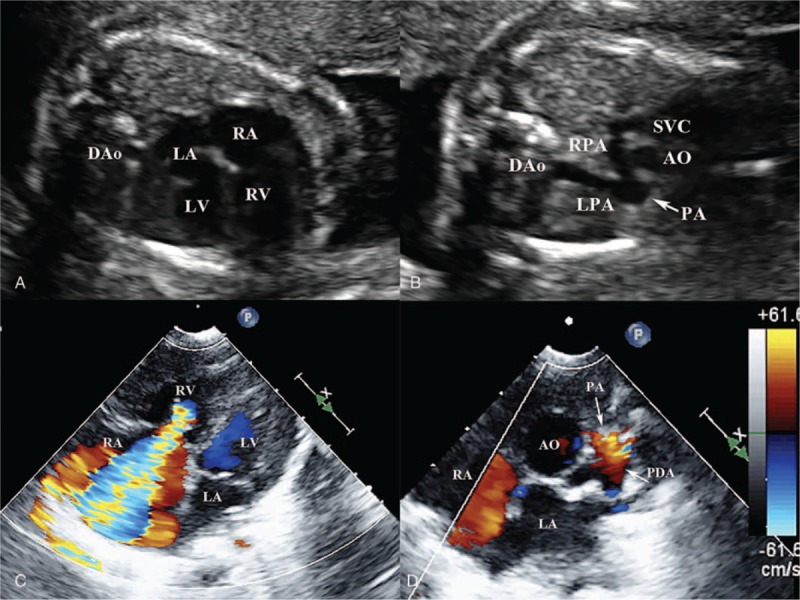
The prenatal and postnatal echocardiography of type I pulmonary atresia with intact ventricular septum. (A) The prenatal echocardiography at 23 weeks’ gestation, four-chamber view shows right ventricle is well developed, no venticulo-coronary artery communication, intact ventricular septum. (B) The 3-vessel-trachea view shows pulmonary atresia (*narrow*). (C) The postnatal echocardiography of the same fetus, four-chamber view shows right ventricle is well developed, severe tricuspid regurgitation, no venticulo-coronary artery communication. (D) Parasternal short axis shows pulmonary atresia (*arrow*), patent ductus arteriosus. AO = aorta, DAo = descending aorta, LA = left atrium, LPA = left pulmonary artery, LV = left ventricle, PA = pulmonary atresia, PDA = patent ductus arteriosus, RA = right atrium, RPA = right pulmonary artery, RV = right ventricle, SVC = superior vena cava, VCAC = ventriculo-coronary artery communication.

**Figure 4:**
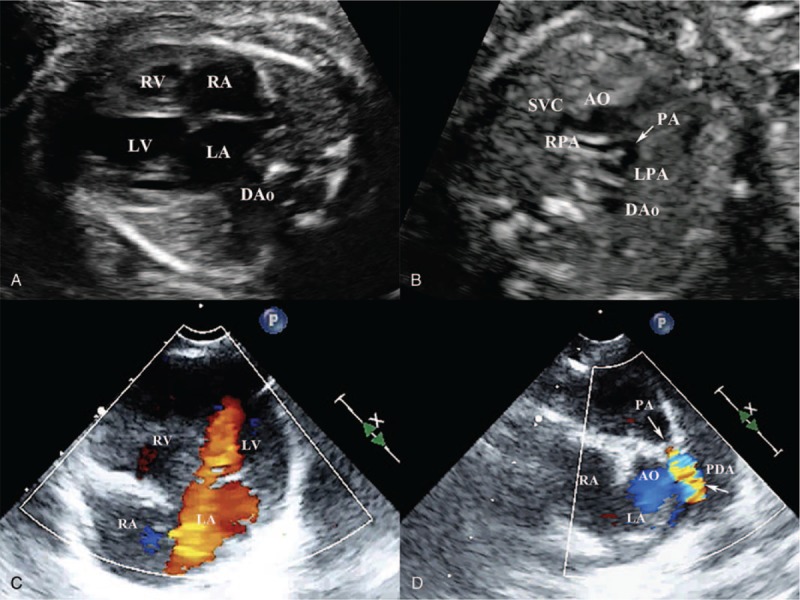
The prenatal and postnatal echocardiography of type II pulmonary atresia with intact ventricular septum. (A) The prenatal echocardiography at 22 weeks’ gestation, four-chamber view shows the absence of right ventricular trabecular portion, no venticulo-coronary artery communication, intact ventricular septum. (B) The 3-vessel-trachea view shows pulmonary atresia (*narrow*). (C) The postnatal echocardiography of the same fetus, four-chamber view shows the absence of right ventricular trabecular portion, no venticulo-coronary artery communication, no tricuspid regurgitation. (D) Parasternal short axis shows pulmonary atresia (*arrow*), patent ductus arteriosus. AO = aorta, DAo = descending aorta, LA = left atrium, LPA = left pulmonary artery, LV = left ventricle, PA = pulmonary atresia, PDA = patent ductus arteriosus, RA = right atrium, RPA = right pulmonary artery, RV = right ventricle, SVC = superior vena cava, VCAC = ventriculo-coronary artery communication.

**Figure 5:**
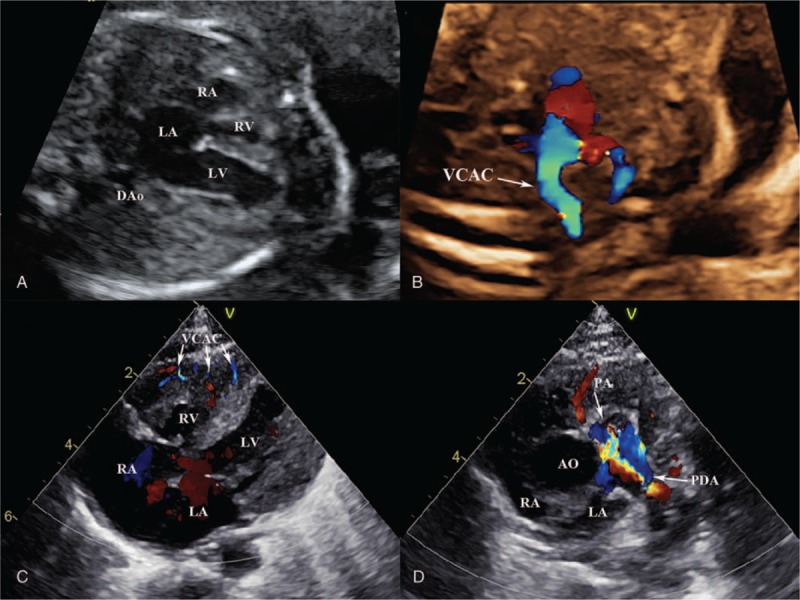
The prenatal and postnatal echocardiography of type III pulmonary atresia with intact ventricular septum. (A) The prenatal echocardiography at 25 weeks’ gestation, four-chamber view shows the absence of right ventricular trabecular portion, intact ventricular septum. (B) Color Doppler shows venticulo-coronary artery communication (*narrow*). (C) The postnatal echocardiography of the same fetus, four-chamber view shows the absence of right ventricular trabecular portion, venticulo-coronary artery communication (*narrow*). (D) Parasternal short axis shows pulmonary atresia (*arrow*), patent ductus arteriosus. DAo = descending aorta, LA = left atrium, LV = left ventricle, PA = pulmonary atresia, PDA = patent ductus arteriosus, RA = right atrium, RV = right ventricle, VCAC = ventriculo-coronary artery communication.

The follow-up data for all the PA/IVS fetuses is shown in Table 1 and Figures 2 to 5. Of the 51 fetuses with PA/IVS, 20 were type I, 17 were type II, and 14 were type III. Only one fetus displayed right aortic arch. The karyotypes of all the fetuses were normal. In total, 29 of the 51 fetuses resulted in live birth and 22 were terminated. Of the live birth fetuses, 13 out of 29 had biventricular repairs, 15 out of 29 had univentricular repairs, including 5 Blalock–Taussig (B–T) shunt operations, 6 Glenn operations, 4 Fontan operations. Only one of the 29 fetuses had a fetal intervention operation. Of the 22 terminated fetuses, one was terminated 3 weeks after fetal intervention.

**Table 1:**
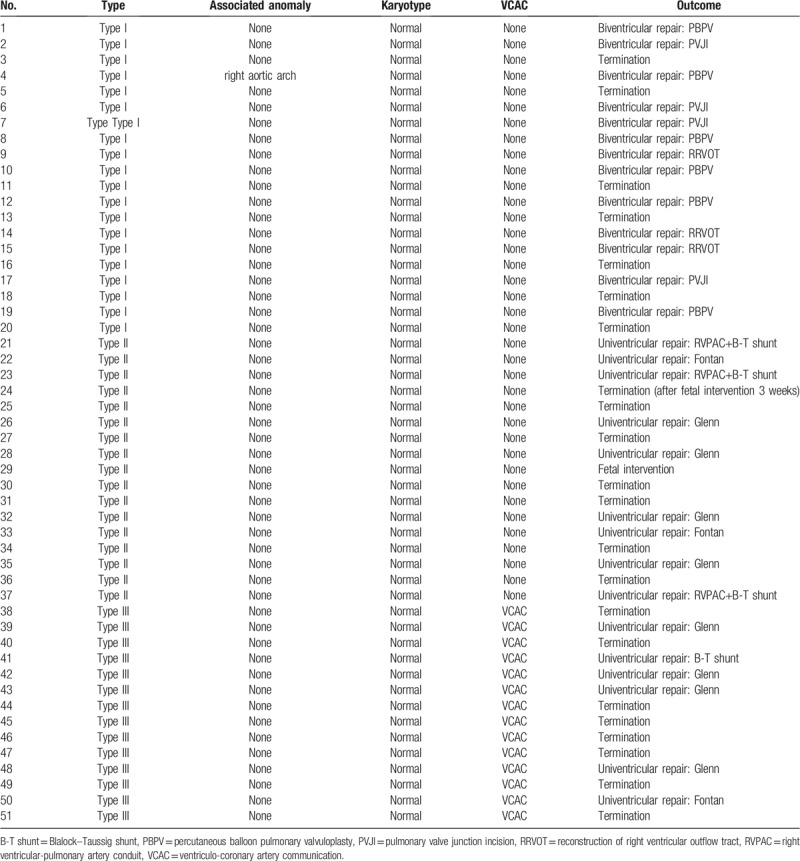
Prenatal diagnosis and follow-up in 51 fetal pulmonary atresia with intact ventricular septum.

Of the type I fetuses, 20 were diagnosed with presence of RV inlet portion, trabecular portion, and infundibulum portion and absence of VCAC; 13 fetuses received biventricular repair and 7 were terminated.

Of the type II fetuses, 17 were diagnosed with the absence of RV trabecular portion, the presence of RV inlet portion, and infundibulum portion but small cavity, the absence of VCAC. Nine fetuses received univentricular repair, including 3 B–T shunt operations, 3 Glenn operations and 3 Fontan operations. Two fetuses had fetal intervention operations, one of which was terminated 3 weeks later. Six fetuses were terminated.

Of the type III fetuses, 14 were diagnosed with the absence of RV trabecular portion and infundibulum, small inlet portion, and VCAC. Six fetuses had univentricular repair, including 2 B–T shunt operations, 3 Glenn operations, and 1 Fontan operation. Eight fetuses were terminated.

### 3.2 Two-dimensional parameters of fetal PA/IVS between biventricular repair and univentricular repair

The results of the two-dimensional parameters between the biventricular and univentricular repair groups are listed in Table 2. Of the 51 fetuses, 28 underwent surgical operations, including 13 (46%) biventricular repairs and 15 (54%) univentricular repairs. There was no difference in gestational age between the biventricular and univentricular repair groups (26.96 ± 5.52 vs 27.74 ± 5.75, *P* = .72). The TV *z*-score was significantly higher for the biventricular repair group than the univentricular repair group (−1.20 ± 0.98 vs −4.33 ± 0.80, *P* = .000), whereas the MV *z*-score was not significantly different between the 2 groups (0.28 ± 0.14 vs 0.26 ± 0.11, *P* = .65). TV/MV and RV/LV length were significantly higher for the biventricular repair group than the univentricular repair group (0.81 ± 0.14 vs 0.54 ± 0.09, 0.71  ± 0.11 vs 0.49 ± 0.09, *P* = .000).

**Table 2:**
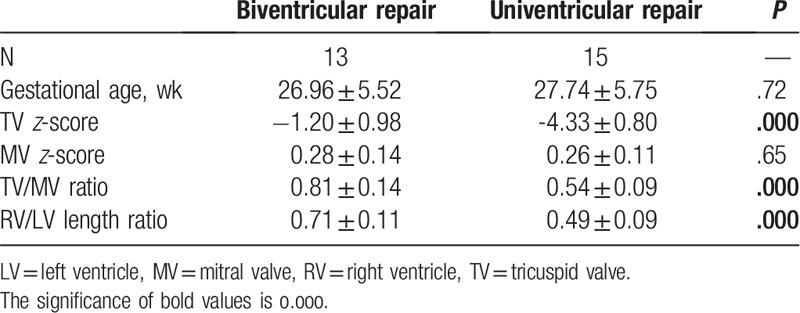
Comparison of fetal two-dimensional echocardiographic parameters between biventricular repair and univentricular repair.

### 3.3 Doppler flow parameters for fetal PA/IVS between the biventricular and univentricular repair groups

The results of the Doppler flow parameters between the biventricular and univentricular repair groups are shown in Table 3. Moderate or severe TR was present in 11 out of 13 (85%) in the biventricular repair group compared with 1 out of 15 (7%) in the univentricular repair group (*P* = .000). None of the biventricular repair group displayed evidence of TR (0/13). VCAC was present in 12 of the 15 fetuses in the univentricular repair group, and was significantly different between the 2 groups (*P* = .000). TID/CCD was significantly higher in the biventricular repair group compared with the univentricular repair group (39.20 ± 3.84 vs 29.16 ± 4.58, *P* = .000). MCA PI and UA PI were not significantly different between the 2 groups (1.94 ± 0.41 vs 2.07 ± 0.31, *P* = .36; 1.27 ± 0.42 vs 1.03 ± 0.19, *P* = .06).

**Table 3:**
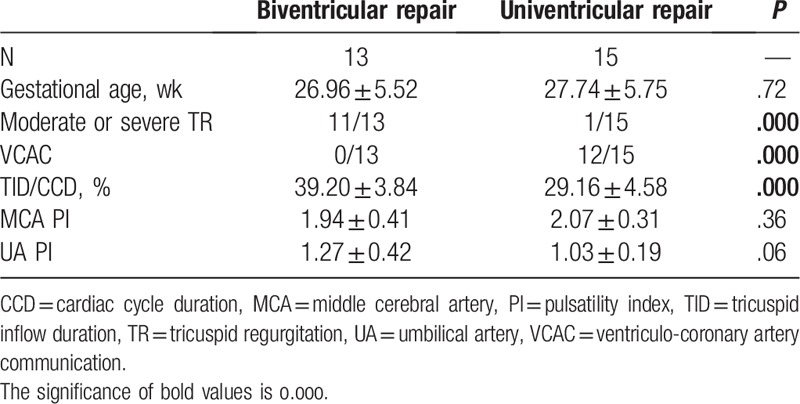
Comparison of fetal Doppler flow parameters between biventricular repair and univentricular repair.

### 3.4 ROC analysis for biventricular repair diagnostic test evaluation

**Figure 6:**
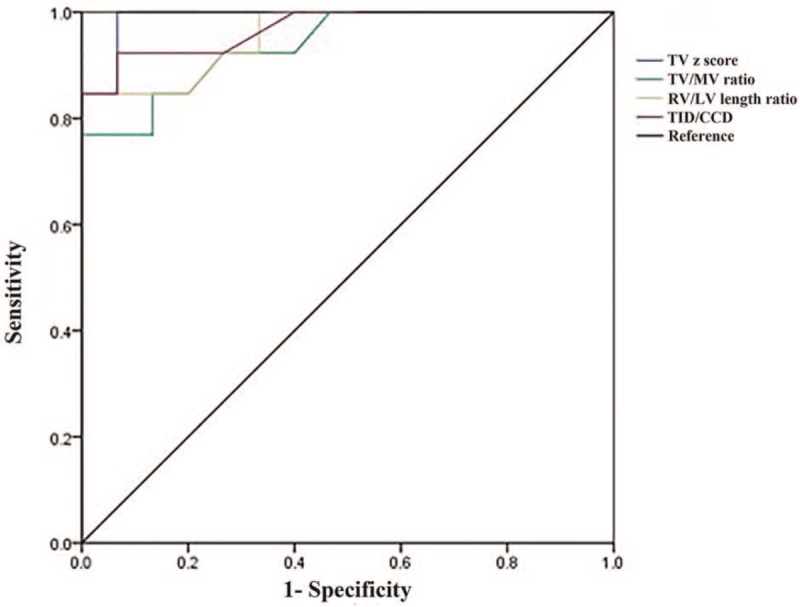
Received operation curves for the TV *z*-score, TV/MV ratio, RV/LV length ratio, and TID/CCD. Biventricular repair characteristic curves yielded areas under the curve of 0.99 for TV *z*-score, 0.94 for TV/MV ratio, 0.96 for RV/LV length ratio, and 0.97 for TID/CCD.

The results of the ROC analysis for biventricular repair diagnostic test evaluation are shown in Table 4 and Figure 6. The biventricular repair characteristic curves yielded AUCs of 0.99 for TV *z*-score, 0.94 for TV/MV, 0.96 for RV/LV length, and 0.97 for TID/CCD. Cutoff values for the biventricular repair characteristic curves were TV *z*-score > −3.28, TV/MV >0.71, RV/LV length >0.62, and TID/CCD >33.95% (*P* < .01 for all). The 95% confidence intervals for TV *z*-score, TV/MV, RV/LV length, and TID/CCD were 0.96 to 1.00, 0.85 to 1.00, 0.89 to 1.00, and 0.91 to 1.00, respectively. The sensitivities of the TV *z*-score, TV/MV, RV/LV length, and TID/CCD were 100%, 77%, 85%, and 92%, respectively. The specificities of the TV z-score, TV/MV ratio, RV/LV length ratio, and TID/CCD were 94%, 100%, 100%, and 94%, respectively.

**Table 4:**
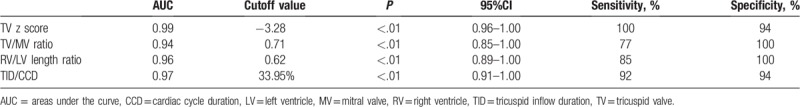
Received operation curves for the TV *z*-score, TV/MV ratio, RV/LV length ratio, and TID/CCD.

## 4 Discussion

PA/IVS is characterised by a continuum from well developed to dysplasia of the RV and TV, the absence or presence of VCAC, and pulmonary atresia. This morphologic diversity results in different outcomes of fetal PA/IVS. Fetal PA/IVS with a good-sized RV and TV and absence VCAC treated by biventricular repair has a much better prognosis compared with those with dysplasia of the RV and TV and presence of VCAC treated by univentricular repair.^[9–11]^ Diagnosis of fetal PA/IVS requires effective prenatal prediction of the cardiac anomaly and consideration of the benefits of biventricular or univentricular repair. Accurate prediction would help parents make decisions, such as whether to terminate or continue the pregnancy and whether to undergo surgery after birth or identify an appropriate fetal intervention that may change the outcome and improve RV growth, such as univentricular or biventricular repair.^[12,13]^

In the present study, we classified fetal PA/IVS according to RV development and the presence or absence of VCAC. In total, 20 fetuses were diagnosed as type I, including 13 that underwent biventricular repair and 7 that were terminated. This group was characterized by well-developed RV and TV and absence of VCAC, and outcomes tended toward biventricular repair. Of the 17 fetuses diagnosed as type II, 9 underwent univentricular repair, 2 had fetal intervention operations, and 6 were terminated. This group was characterized by RV and TV dysplasia, the absence VCAC, and outcomes tended toward univentricular repair, although fetuses also underwent fetal intervention operations to increase the RV antegrade flow to the pulmonary artery to promote the development of RV, enabling biventricular repair. A total of 14 fetuses were diagnosed as type III, including 6 that underwent univentricular repair and 8 that were terminated. This group was characterized by RV and TV dysplasia and the presence of VCAC. Echocardiographic classification of fetal PA/IVS enables variable degrees of pathology and pathophysiology of RV, TV, and VCAC to be determined.

We aimed to identify predictors of PA/IVS outcome to provide reliable prenatal counseling. Only one fetus with type I displayed right aortic arch, and the karyotype of all the fetuses were normal. PA/IVS is rarely associated with extra cardiac anomalies and chromosomal abnormalities.^[14]^ Aggarwal et al^[15]^ reported a patient with PA/IVS, confluent pulmonary arteries supplied by an arterial duct and chromosome 22q11.2 microdeletion. Although associated anomalies and genetic abnormalites are rare, it is important to be aware of this association. The tricuspid *z*-score was significantly higher for the biventricular repair group than the univentricular repair group (*P* = .000), whereas the mitral *z*-score was not significantly different between 2 groups (*P* = .65). Both TV/MV and RV/LV length were significantly higher in the biventricular repair group compared with the univentricular repair group (*P* = .000). These values indicated a close relationship between TV and RV, and a well-developed TV was more likely to have a better RV. There was significant difference between the biventricular and univentricluar repair groups for moderate or severe TR and VCAC. Moderate or severe TR indicated the presence of a well-developed TV annulus and the RV cavity bears the force of ventricular filling leading to significant regurgitation. VACA was present in type III, but absent in type I and type II, and none of fetuses underwent biventricular repair in type III of PA/IVS. It is important to recognize VCAC by examining the small RV cavity, RV wall, and septum. Delineating the details of fetal coronary artery stenosis or atresia that result in RV-dependent coronary circulation is challenging and should be performed by postnatal evaluation using cardiac catheterization. There were no subsequent attempts to connect the RV to the pulmonary artery to decompress the RV pressure because RV decompression may lead to RV “steal” in the presence of fistulas alone and ischaemia, coronary isolation, or myocardial infarction in the presence of coronary stenosis.^[16–18]^

Our study identified several fetal echocardiographic makers predictive of postnatal biventricular repair versus univentricular repair. The cutoff values that predicted biventricular repair were TV *z*-score > −3.28, TV/MV >0.71, RV/LV length >0.62, and TID/CCD >33.95%. The sensitivities of the TV *z*-score, TV/MV, RV/LV length, and TID/CCD were 100%, 77%, 85%, and 92%, respectively. The specificities of the TV *z*-score, TV/MV, RV/LV length, and TID/CCD were 94%, 100%, 100%, 94%, respectively. Many studies have reported that a TV *z*-score value of −3 is an important discriminator between biventricular and univentricular repair strategy.^[19–22]^ In the present study, TV *z*-score of the 3 fetuses that underwent biventricular repair was approximately −3.13 to −3.21, which is useful to predict biventricular or univentricular repair. Higher TV/MV and RV/LV indicate a postnatal biventricular repair. TID implies RV filling and provides information about TV function, RV volume and compliance, whereas higher TID/CCD indicates a well-developed RV cavity.

The limitation of this study was a relatively small sample size. Postnatal surgical strategy may have been influenced by an individual surgeon and practice experience in our center, and it is possible that a patient would undergoing biventricular repair in our institution may have undergone univentricular repair elsewhere.

In conclusion, fetal echocardiography can classify PA/IVS based on variable degrees of TV, RV, and VCAC. We identified TV *z*-score, TV/MV, RV/LV length, TID/CCD, moderate and severe TR and VCAC as important variables that differentiate between biventricular and univentricular repair strategies. These findings may have implications for prenatal counseling and predict fetal outcome.

## Acknowledgments

We are grateful to the staff at the Department of Ultrasound, Gene Center, and Children's Heart Center.

## Author contributions

**Data curation:** Yanan Li, Ying Wang, Bangtian Peng.

**Formal analysis:** Hongdan Wang.

**Funding acquisition:** Lin Liu, Yanan Li.

**Investigation:** Cunying Cui, Yuanyuan Liu.

**Methodology:** Taibing Fan.

**Project administration:** Lin Liu.

Lin Liu orcid: 0000-0001-8973-8738.
